# A Duoethnographic study of professional identity negotiation by two returned art theory teachers in Chinese higher education

**DOI:** 10.3389/fpsyg.2026.1922669

**Published:** 2026-09-01

**Authors:** Sijie Fan, Jianwei Wei, Tingting Wen

**Affiliations:** 1 Guangdong-Hong Kong-Macau Great Bay Area Shipping Research Institute, Guangzhou Maritime University, Guangzhou, China; 2 College English Teaching Department, Guangdong University of Foreign Studies South China Business College, Guangzhou, China; 3 Rao Zongyi Culture Research Institute, Shenzhen University, Shenzhen, China

**Keywords:** art theory education, duoethnography, reflective practice, returned academics, teacher identity

## Abstract

**Introduction:**

Internationalization requires returned academics to reconsider pedagogical commitments developed across different educational contexts. This study explored how two returned university art theory teachers in southern China negotiated professional identities while adapting their teaching to changing classroom conditions.

**Methods:**

A duoethnographic design brought one teacher’s contemporaneous reflections into dialogue with the other’s retrospective accounts of comparable early-career experiences. Dialogues, reflective accounts, observation notes, and teaching materials were examined through reflexive thematic analysis.

**Results:**

The analysis identified learner, explorer, and pedagogical enactor as overlapping identity positions that the teachers entered and revisited through reflection and practice. Classroom responses unsettled existing assumptions and prompted further inquiry into theoretical explanation, participation, and student agency. Through dialogue, the teachers developed more contextually responsive approaches that combined conceptual scaffolding with opportunities for discussion and culturally situated interpretation.

**Discussion:**

The findings present professional identity negotiation as a recursive and situated process in which dialogic reflection connected teacher learning with pedagogical decision making. Farrell’s reflective domains helped interpret how the teachers’ concerns connected educational purposes, pedagogical commitments, classroom practices, and wider sociocultural conditions. The study provides a context-specific account of how two returned art theory teachers reconsidered overseas pedagogical commitments in Chinese higher education.

## 1 Introduction

Internationalization has brought different educational histories, pedagogical expectations, and understandings of knowledge and participation into closer contact within higher education (Qiu et al., 2024). Within Chinese higher education, these encounters are evident in the experiences of academics who return after studying overseas (Ai and Wang, 2017). Returned teachers may draw on pedagogical commitments developed across educational contexts while responding to the students, disciplines, and institutional conditions they encounter. This process involves more than the direct transfer of teaching practices. It may require teachers to reconsider how discussion, authority, participation, and student agency are understood and enacted in particular classrooms, reflecting the situated negotiation of transcultural teacher identity (Soong et al., 2021). Such questions can be especially significant during early career teaching, when professional identity and pedagogical confidence remain closely connected with everyday classroom experience (Christensen and Weissmann, 2025).

These concerns have particular relevance to university art theory teaching. Higher art education brings aesthetic concepts, art history, visual culture, and critical interpretation into relation with students’ social and cultural environments (Wang et al., 2025). Research in arts and design education has also emphasized the importance of connecting conceptual learning with situated practices and contemporary concerns (Kang et al., 2025). In art theory classrooms, visual and cultural materials may provide objects through which theoretical ideas can be examined, applied, and questioned. Their educational significance, however, depends on how teachers frame the materials, structure participation, and create opportunities for interpretation in relation to particular students and classroom contexts.

Teacher identity is continually negotiated through professional experience, reflection, and interaction with changing educational contexts (Sahling and De Carvalho, 2021; Guo and Sidhu, 2024). This negotiation is closely connected with teacher learning, as pedagogical decisions develop through the interplay of personal histories, classroom experience, and institutional conditions (Wang et al., 2021; Vermunt et al., 2023). For returned art theory teachers, these processes converge as they reconsider pedagogical commitments developed overseas in relation to particular students, disciplinary purposes, and institutional settings. Their experiences provide a specific context for examining how identity negotiation and pedagogical adaptation develop together through dialogue about classroom practice.

Against this background, the present study draws on a duoethnography of two returned university art theory teachers working at different universities in southern China. The first author began university teaching 1 year before the third author, and the study commenced during the third author’s first semester of teaching. Their dialogue brought contemporaneous reflections on current teaching into relation with retrospective accounts of comparable experiences, while the second author served as a methodological critical friend who questioned interpretations and supported analytical scrutiny. This design drew on duoethnography’s emphasis on difference, mutual questioning, and the reinterpretation of experience (Norris and Sawyer, 2012). Farrell’s (2020) reflective domains provided a conceptual vocabulary for interpreting the concerns discussed by the teachers. Focusing on these two cases, the study examines how professional identity was negotiated through dialogic reflection and how developing understandings informed pedagogical decisions across changing classroom conditions. It offers a situated account of learner, explorer, and pedagogical enactor as overlapping identity positions and considers how the teachers adapted theoretical explanation, scaffolding, participation structures, and visual or cultural materials for particular student groups.

## 2 Literature review and theoretical framework

### 2.1 Teacher identity in cross-cultural higher education

Post-structural perspectives conceptualize identity as historically situated, relationally produced, and continually performed within social discourse, shifting attention from stable professional categories to the ways teachers position themselves through pedagogical action, institutional language, and cultural encounter (Hall, 1992; Butler, 1990). In teacher education, this perspective has encouraged scholars to read pedagogy as an identity practice, with teachers’ decisions about authority, participation, curriculum, and assessment expressing who they are becoming in relation to learners, knowledge, and professional communities (Tucker, 2025).

The internationalization of higher education intensifies this identity work by placing teachers within overlapping cultural and institutional expectations. Some returned academics report pedagogical commitments shaped by their overseas learning experiences, including greater emphasis on student agency, discussion, and interpretive autonomy, while their subsequent teaching may unfold in Chinese university contexts shaped by different institutional arrangements, participation norms, and educational histories (Ai and Wang, 2017; Soong et al., 2021; Qiu et al., 2024). Their professional transition involves movement across educational systems, career stages, and epistemic expectations, which can create uncertainty around what counts as legitimate teaching and credible academic identity (Dong et al., 2025).

Teacher learning research deepens this discussion by showing that professional growth develops through changing relations among personal histories, contextual demands, and learning outcomes (Vermunt et al., 2023). Teachers who enter new professional roles often need to balance disciplinary expertise, prior professional experience, and emerging pedagogical responsibilities, a process that can generate both identity tension and professional renewal (Antera and Teräs, 2024; Bükki and Fehérvári, 2024). In early academic careers, reflection on everyday teaching events can make visible how identity develops through practice, emotion, and institutional positioning (Christensen and Weissmann, 2025).

### 2.2 Teacher professional development and relational professional learning

Teacher professional development has been discussed through motivation, leadership, empowerment, and institutional support, with particular attention to how teachers maintain long term engagement with professional learning under changing educational conditions (Zhang et al., 2021; Bas, 2026). Leadership for learning and organizational structures can shape the conditions under which teachers participate in development activities, while continuity in professional learning depends on teachers’ capacity to interpret practice, exercise agency, and adapt pedagogical judgment to specific contexts (Karakose et al., 2026; Wang et al., 2021).

Relational approaches to professional development are especially relevant in higher education, where teachers often learn through curriculum dialogue, peer observation, collaborative inquiry, and reflective exchange. Staff student partnership research suggests that curriculum development can redistribute agency and make teaching design more responsive to learner experience (Boyle et al., 2024). For teachers working across cultural expectations, relational professional learning can create spaces where anxiety, uncertainty, and classroom difficulty become shared materials for professional inquiry (Sahling and De Carvalho, 2021; Christensen and Weissmann, 2025).

### 2.3 Culturally situated art theory pedagogy

Higher education scholarship increasingly connects cultural, artistic, curricular, and professional dimensions of educational change. Design thinking and problem-based learning have been examined for their support of interdisciplinary dialogue, collaborative creation, and active learning in teacher education (Heystek et al., 2025). Arts and design education research further shows that student motivation, creative practice, and future oriented imagination can shape learning in art and design contexts (Kang et al., 2025).

Cultural sustainability concerns the continued reinterpretation of traditions, collective memory, aesthetic values, and cultural identity under conditions of social change. This concern connects with resilience, belonging, and the capacity of academic communities to maintain meaningful cultural knowledge across institutional pressures (Rodriguez Castro et al., 2026). Within art theory education, aesthetics, art history, visual culture, and critical theory can be brought into relation with Eastern cultural traditions, intangible cultural heritage, and contemporary cultural identity, potentially creating opportunities for inherited cultural resources to become objects of interpretation, critique, and creative renewal (Wang et al., 2025).

This connection between art theory education and culturally situated interpretation places distinctive demands on teachers. Art theory teachers are expected to introduce global theoretical languages while also guiding students toward culturally situated interpretation, a task that requires sensitivity to visual experience, cultural memory, and student agency (Kang et al., 2025; Wang et al., 2025). Classroom activities connecting theory with heritage, visual culture, and creative inquiry may create opportunities for students to interpret cultural resources through analytic, creative, and dialogic activity (Rodriguez Castro et al., 2026).

### 2.4 Reflective practice and duoethnographic inquiry

Reflective practice offers an analytical route for examining how teachers interpret experience, question assumptions, and reshape professional action in relation to changing educational contexts. Farrell’s (2020) framework identifies five interconnected domains of reflection known as philosophy, principles, theory, practice, and beyond practice. These domains offer complementary points of attention rather than a hierarchy or fixed developmental sequence.

Philosophy concerns teachers’ understandings of educational purposes, knowledge, and professional identity. Principles refer to the values and assumptions guiding pedagogical decisions. Theory addresses the explanations teachers use to plan and justify particular approaches, while practice focuses on classroom actions and the relationship between stated beliefs and enacted teaching. Beyond practice extends reflection to the social, cultural, and institutional conditions shaping teachers’ work (Farrell, 2020). Together, the domains enable reflection to move between professional beliefs, pedagogical reasoning, classroom experience, and the wider contexts of teaching.

Duoethnography adds a relational dimension to reflective inquiry by bringing different professional histories and interpretations into dialogue (Norris and Sawyer, 2012). Difference and mutual questioning can make familiar explanations open to reconsideration rather than merely confirm existing understandings (Breault, 2016). This orientation is relevant to cross-cultural teacher identity research, where interpretations of current teaching may be shaped by overseas learning, professional memory, and locally situated classroom experience (Guo and Sidhu, 2024).

In this study, the two perspectives served distinct but complementary purposes. Duoethnography informed the relational examination of experience, while Farrell’s (2020) framework provided a lens for interpreting the domains addressed in the teachers’ reflections. The framework was not used as a predetermined coding structure and did not generate the identity positions reported in the findings.2.5 Research Gap and Research Questions.

Existing scholarship leaves two connected gaps. On the one hand, studies of returned academics have mainly examined career transition, institutional integration, and internationalization, with limited attention to how returned teachers in art theory negotiate professional legitimacy through everyday classroom reflection (Ai and Wang, 2017; Dong et al., 2025). On the other hand, research on art and design education has addressed creative practice, aesthetic education, student motivation, and curriculum design, while the teacher identity work that turns cultural resources into pedagogical resources for art theory learning remains underdeveloped (Kang et al., 2025; Rodriguez Castro et al., 2026). These two gaps intersect in the underexplored process through which returned art theory teachers reinterpret overseas pedagogical values within particular Chinese higher education contexts and connect reflective professional learning with culturally situated art theory pedagogy. Less is known about how such identity negotiations unfold through sustained dialogue between returned teacher-researchers, particularly when current classroom experiences are juxtaposed with retrospective professional memories.

These connected gaps matter for higher education teaching and academic development. Art theory teachers work at the intersection of global knowledge, Eastern cultural traditions, institutional expectations, and student learning histories. Their professional identities influence how they understand participation, select cultural materials, adapt pedagogical theories, and create opportunities for students to reinterpret heritage and visual culture (Vermunt et al., 2023). A focus on reflective identity work can therefore clarify how culturally situated teaching takes shape in cross-cultural art theory classrooms.

Accordingly, the study proposes the following questions.

- What identity positions emerged as the two returned art theory teachers reflected on their cross-cultural teaching experiences?
- How did dialogic reflection connect teacher identity, teacher learning, and culturally situated art theory teaching in the two cases?

## 3 Materials and methods

### 3.1 Research design

This study employed duoethnography to examine how two returned art theory teachers negotiated their professional identities and pedagogical commitments in Chinese university classrooms (Guo and Sidhu, 2024). Duoethnography treats lived experience as a relational site of inquiry in which researchers juxtapose personal histories, memories, and interpretations to question familiar understandings of a shared phenomenon (Norris and Sawyer, 2012). Rather than seeking a unified account, it uses difference, questioning, and mutual interruption as analytic resources (Breault, 2016).

The focal teacher-researchers brought related yet temporally distinct professional experiences into the inquiry. Manxin reflected on current classroom events, while Juzi revisited comparable experiences from her preceding year of teaching (Norris and Sawyer, 2012). Juzi’s earlier experience was not treated as a model for Manxin to follow. It served as a comparative narrative resource that Manxin could question when interpreting current events. Manxin’s experiences also prompted Juzi to reconsider some of her earlier explanations. Meaning was therefore developed through comparison, disagreement, and the revision of both researchers’ interpretations (Norris and Sawyer, 2012).

The design brought together classroom events, professional emotions, overseas learning memories, institutional expectations, and teaching decisions as materials for identity work. It supported an understanding of teacher identity as relational and situated rather than as a stable attribute or linear developmental process (Hall, 1992; Tucker, 2025). Reflexive thematic analysis was used within this design to examine both what the teachers said and how interpretations changed across dialogic exchanges (Trainor and Bundon, 2021).

### 3.2 Research context and participants

The study was conducted from June 2024 to June 2025 in two universities in a large city in southern China. The focal duoethnographic participants were the first author, referred to by the pseudonym Juzi, and the third author, referred to by the pseudonym Manxin. Both were female returned art theory teachers with overseas study experience. They worked at different universities, and Juzi entered university teaching 1 year earlier than Manxin.

The study focused primarily on Manxin’s first academic year of art theory teaching. During the first semester, she taught students beginning the systematic study of art theory. During the second semester, she taught first-year art majors who were more willing to discuss artistic practice but did not necessarily engage readily with theoretical texts. The change in student group provided an opportunity to examine how previously developed teaching understandings were reconsidered under new classroom conditions.

Juzi contributed retrospective accounts from her earlier teaching, participated in the ongoing dialogues, and completed six non-participant observations of Manxin’s classes. She was not positioned solely as a mentor. Manxin’s current experiences also led Juzi to revise her understanding of her own earlier teaching. The two teachers were therefore treated as co-researchers whose interpretations could both be questioned through dialogue (Norris and Sawyer, 2012).

The second author served as a methodological critical friend outside the focal pair (Breault, 2016). This author reviewed developing categories and themes, questioned potentially linear interpretations of identity development, and examined whether claims extended beyond the teacher-generated evidence (Trainor and Bundon, 2021). The role was particularly important in checking whether Farrell’s framework predetermined the findings and whether observations of classroom participation were being presented as evidence of student learning outcomes.

### 3.3 Data collection

Data were generated over approximately 1 year through reflective dialogue, writing, teaching documentation, and classroom observation. Before Manxin began teaching, Juzi and Manxin each prepared a self-narrative about their overseas learning, early teaching experiences, and assumptions about art theory education (Norris and Sawyer, 2012).

The two teachers subsequently conducted 15 formally scheduled reflective dialogues between June 2024 and June 2025. These produced approximately 19 h of recorded conversation. The dialogues were guided by flexible prompts concerning recent classroom events, emotional responses, alternative interpretations, pedagogical decisions, and points of disagreement (Norris and Sawyer, 2012). They were intended to encourage relational questioning rather than consensus (Breault, 2016).

All recorded dialogues were transcribed and checked against the recordings by the three authors. Repetitions and verbal fillers were removed according to the transcription protocol, while the sequence and meaning of the conversational turns were retained. Excerpts selected for publication were translated from Chinese into English and checked against the original transcripts by all three authors. Approximately 24,000 words of dated online communication provided additional records of immediate responses, lesson-planning decisions, and developing interpretations. Reflective journals documented key events such as classroom silence, changes in teaching support, the use of cultural materials, and renewed professional uncertainty.

Juzi conducted six non-participant classroom observations across the two semesters. Each observation covered two consecutive 45-min periods, producing 12 observed class periods. The notes focused on teacher actions and non-identifiable classroom processes such as waiting time, participation formats, group organization, scaffolding, and teacher intervention. Descriptive records were separated from interpretive comments so that observable events were not conflated with assumptions about students’ intentions.

Teaching documents included course syllabi, 33 lesson plans, activity designs, and revision records. They documented changes in classroom questioning, conceptual explanation, scaffolding, grouping, campus visual culture projects, art criticism, curatorial activities, and cultural heritage tasks. Earlier and revised versions were retained when available so that changes could be traced in relation to reflective dialogues and classroom events.

Transcripts and reflective documents were managed in Microsoft Word. Microsoft Excel was used to maintain the data index, code definitions, category comparisons, theme versions, and links between analytic claims and data sources. Files were stored in an access-restricted folder on the university’s Microsoft OneDrive for Business system. Institutional names, course codes, and other identifying information were removed or generalized.

Students were not recruited as research participants, and the analysis was therefore limited to teacher-generated data and non-identifiable classroom observations.

### 3.4 Data analysis

The study used a theoretically informed form of reflexive thematic analysis. Analysis was treated as an iterative and interpretive process in which themes were developed through sustained engagement with the data rather than discovered as objective categories (Trainor and Bundon, 2021). Farrell’s (2020) reflective practice framework informed the study conceptually but was not used as a predetermined coding template.

Juzi and Manxin first read the complete dataset and coded classroom events, professional emotions, interpretations, dialogic challenges, and pedagogical responses. Both researchers reviewed all data sources. Focused separate coding was then conducted on 11 key dialogue transcripts, all six observation notes, 12 lesson plans, and related activity documents. The purposive corpus captured the classroom difficulties, interpretive shifts, and pedagogical changes most directly related to the research questions. Detailed source identifiers and illustrative coding pathways are provided in Appendix A.

Separate coding was used to make interpretive differences visible rather than to calculate intercoder reliability. Reflexive thematic analysis recognizes the researcher as an active interpreter and does not treat complete agreement as the primary indicator of quality (Trainor and Bundon, 2021). When Juzi and Manxin interpreted an event differently, they returned to the full conversational sequence, relevant observation notes, online exchanges, and teaching documents. Interpretations with broader support across the dataset were developed further, while unresolved differences were retained when the available evidence did not justify a single explanation.

The second author served as a methodological critical friend outside the focal pair (Breault, 2016). The second author reviewed developing categories and themes, questioned linear interpretations of professional growth, and examined whether claims extended beyond the available evidence. This process did not remove researcher subjectivity. It made the assumptions and interpretive decisions shaping the analysis more visible (Trainor and Bundon, 2021).

Related codes were progressively combined into categories and candidate themes. During theme development, the authors compared chronological, strategy-based, and identity-based ways of organizing the findings. The identity-based structure was retained for its greater capacity to explain changes in professional interpretation across the two semesters. Temporal developments and pedagogical strategies were retained as evidence within the themes rather than treated as separate result structures.

The identity positions emerged during the movement from codes and categories to candidate themes. They were not established before coding and were not terms repeatedly used by Juzi or Manxin. Learner, explorer, and pedagogical enactor were second-order analytic labels developed by the authors. Re-entering the learner position described recursive movement under changed classroom conditions rather than a fourth stable identity or a return to the original starting point. The positions could overlap, and neither teacher followed a fixed sequence (Tucker, 2025).

Farrell’s (2020) domains of philosophy, principles, theory, practice, and beyond practice were introduced after the candidate themes had been developed. They were used to examine the levels at which reflection occurred and to connect identity movement with teacher learning and pedagogical action. The framework therefore supported the interpretation of the themes but did not generate the identity positions.

The analysis was adapted to duoethnography by attending to dialogic sequences rather than treating each statement as an isolated unit. The researchers examined how questions, disagreements, comparisons of past and present experience, and responses from one teacher altered or complicated the other’s interpretation (Norris and Sawyer, 2012). Differences between the two accounts were treated as analytic resources rather than obstacles to a unified narrative (Breault, 2016).

Following Trainor and Bundon (2021), themes were retained when they were coherent, distinguishable, supported across data sources, and relevant to the research questions. Uneven outcomes and instances in which reflection did not produce successful change were also examined. The resulting model was treated as a situated interpretation of these two cases rather than a universal developmental sequence (Norris and Sawyer, 2012; Tucker, 2025).

## 4 Results

The analysis generated three identity positions known as learner, explorer, and pedagogical enactor. These positions described how Juzi and Manxin interpreted professional uncertainty, examined pedagogical alternatives, and translated developing understandings into classroom action. Rather than fixed stages or consistent self-descriptions, they were analytic labels developed from recurring relationships among professional emotion, dialogic reflection, and pedagogical decision making. The positions overlapped, and both teachers moved among them as classroom conditions changed. The data-source identifiers cited in the findings are explained in Appendix A.

### 4.1 Entering the learner position

The learner position first emerged through uncertainty about professional legitimacy. During her initial weeks of teaching, Manxin introduced project-based activities that invited students to interpret visual materials, discuss aesthetic concepts, and connect theory with personal experience. She had expected these activities to encourage open discussion. Instead, she observed extended silence, limited voluntary responses, and a tendency for students to wait for further explanation. She initially interpreted this response as evidence that she might not be able to translate her overseas educational experience into effective teaching in a Chinese university classroom: “After the students fell silent, I almost immediately concluded that I did not know how to teach. I then realized that student silence was an observation, whereas waiting for a standard answer and lacking teaching ability were interpretations” (RJ-M02).

Juzi questioned whether visible silence necessarily indicated disengagement or teaching failure. She asked Manxin to distinguish what she had observed from the explanations she had attached to it. Their discussion identified several possibilities, including unfamiliar task structures, limited preparation time, concern about speaking publicly, and Manxin’s tendency to provide an answer soon after asking a question. Juzi’s first observation notes also recorded differences among individual writing, small-group discussion, and whole-class speaking. Some students who did not respond publicly had written notes or participated in peer exchanges. These records did not establish why students remained silent, but they complicated Manxin’s initial interpretation of silence as general non-participation.

The dialogue also unsettled Juzi’s retrospective account. Juzi had previously attributed limited participation in her own classes partly to students’ dependence on teacher direction. Reconsidering her experience alongside Manxin’s current classroom events drew attention to the design of the activities and to her expectation that students would readily adopt discussion practices familiar from her overseas study. Juzi therefore began to question an explanation that located the difficulty mainly in students’ prior learning experiences.

The learner position involved this shift from immediate self-judgment or student-focused explanation toward closer examination of classroom conditions. Manxin continued to regard students’ previous educational experiences as relevant, while Juzi increasingly emphasized task wording, waiting time, participation format, and the risks associated with public interpretation. Their accounts did not become identical. Manxin maintained that *“students’ previous learning habits remained an important reason for the silence”*. Juzi responded that “*task demands, the risk of public expression, and the teacher’s handling of waiting may have operated together*”. They agreed to retain rather than prematurely resolve this difference (DG-04).

This process changed how both teachers understood professional learning. Uncertainty did not disappear, but it became an object of inquiry rather than solely a sign of inadequate knowledge or authority. The learner position therefore referred to their willingness to reconsider the assumptions through which they interpreted classroom events.

### 4.2 Entering the explorer position

The explorer position developed as the teachers used their revised interpretations to examine alternative forms of participation and support. Manxin did not abandon project-based learning, but she changed how students entered the activities. She introduced visual examples, model interpretations, individual preparation, and more explicit discussion prompts before asking students to make theoretical claims. Her lesson-plan revisions documented a shift from open invitations to more sequenced tasks that connected observation, conceptual explanation, peer discussion, and public response (LP1-05).

This adjustment initially produced another tension. Manxin associated increased teacher explanation with teacher-centered instruction and worried that she had compromised her commitment to student agency. She explained, *“I used to equate more explanation with compromising student-centered teaching. I now think the question is what happens after the explanation. If students can use a concept to reconsider the material, the explanation may provide necessary preparation”* (DG-05). Juzi responded, *“We cannot judge student-centeredness only by how long the teacher speaks. We need to examine whether the explanation closes the question or provides resources for subsequent interpretation”* (DG-05). Manxin subsequently retained direct explanation but followed it with tasks requiring students to apply, compare, or question the concepts introduced.

Juzi’s remembered experience provided a contrasting case. During her first year of teaching, she had reduced scaffolding after becoming concerned that students were relying too heavily on teacher guidance. Several groups subsequently approached the activity superficially or waited for further direction. Reflecting on this experience, Juzi explained, *“I had mistaken autonomous learning for teacher withdrawal. I removed the visible constraints without helping students develop ways of generating questions, collaborating, or judging the quality of materials”* (DG-06). She later acknowledged that her attribution of the difficulty to students’ limited autonomy *“may have arisen partly from inadequate instructional design”* and that Manxin’s current experience had changed how she understood her past teaching (RJ-J02).

This reinterpretation affected Manxin’s subsequent planning. Rather than treating teacher support and student agency as opposing choices, she varied support according to the demands of the task. Revised materials provided conceptual prompts and procedures at the beginning of an activity while leaving the selection of examples and the development of interpretations more open. The dialogue did not establish one ideal amount of scaffolding. It helped the teachers ask what particular forms of support enabled students to undertake interpretive work.

Exploration also involved examining how group organization shaped visible participation. Observation notes indicated that students in familiar peer groups often began tasks more quickly, although familiarity did not ensure equal participation. In some groups, one member continued to dominate the discussion. Manxin therefore combined familiar grouping with individual preparation, rotating roles, and a designated questioner. These changes reflected an exploratory process in which an initial adjustment was examined for both its possibilities and its limitations (DG-07, LP1-12).

The explorer position was thus characterized by provisional action and continued questioning. Juzi’s earlier decisions were not presented as solutions for Manxin to copy. They became comparative cases through which both teachers reconsidered the purposes and consequences of explanation, scaffolding, and group organization.

### 4.3 Entering the pedagogical enactor position

The pedagogical enactor position emerged when the teachers connected their developing interpretations with more contextually responsive activity designs. This position did not indicate that uncertainty had ended or that the teachers had reached a final level of professional development. It described moments in which reflection became visible in coordinated decisions about content, participation, conceptual support, and classroom roles.

Manxin increasingly used materials connected with students’ immediate visual and cultural environments. Activities invited students to examine campus visual culture, regional art forms, curatorial practices, and intangible cultural heritage through art theory concepts. The use of cultural materials also prompted the teachers to reconsider the intended outcomes of these activities. Manxin acknowledged, “I originally described the objective as enhancing students’ cultural identification. I now see that this predetermined the expected conclusion and exceeded what our teacher-generated data could demonstrate” (DG-07). Juzi proposed that the task should allow students to examine how heritage was selected, displayed, commercialized, or reinterpreted rather than require a predetermined attitude (DG-07).

In one series of activities, students were assigned roles such as curator, art critic, and cultural interpreter. Manxin provided theoretical concepts and examples before asking groups to develop exhibition interpretations or short critical responses. The activity required students to consider historical context, modes of display, intended audiences, omitted perspectives, and possible alternative interpretations (ICH-01). Her records described more visible discussion in some groups when the objects of analysis were recognizable and the expected outputs were clearly structured. However, participation remained uneven, and the teacher-generated data could not establish how students understood the cultural or theoretical significance of the tasks. The finding therefore concerns Manxin’s pedagogical redesign and her interpretation of classroom participation rather than demonstrated student learning outcomes.

Juzi’s retrospective experience contributed to the redesign, but the dialogue also changed Juzi’s interpretation of her own teaching. She came to view her earlier unsuccessful withdrawal of scaffolding less as evidence of students’ limited autonomy and more as a problem involving the relationship among task structure, conceptual preparation, and participation expectations. Her remembered difficulty thereby became a resource for current planning while remaining open to reinterpretation.

The pedagogical enactor position brought together the teachers’ educational purposes, assumptions about participation, understanding of pedagogical approaches, and classroom decisions. Manxin was able to enact a more contextually responsive design without treating either overseas pedagogy or existing classroom practices as a complete model. At the same time, observation and dialogue continued to identify uneven participation and moments when she resumed greater control. Pedagogical enactment was therefore situated and provisional rather than evidence of completed professional transformation.

### 4.4 Moving recursively across identity positions

Manxin’s second semester demonstrated that the identity positions did not form a stable sequence. Her new students were generally more willing to discuss their artistic experiences, but she found it difficult to sustain their engagement with abstract concepts and theoretical texts. The visible willingness to speak that she had previously sought did not necessarily produce the form of theoretical engagement she expected. This change disrupted the confidence developed during the first semester.

Manxin initially attributed the difficulty to inadequate theoretical knowledge. She explained, *“The classroom appeared active, but discussion became unfocused when I asked students to define concepts or justify their claims. I began to wonder whether my theoretical explanations were inadequate”* (DG-10). Juzi responded that the problem might not be how much theory Manxin knew. Instead, theory might not yet have become a tool students needed to address questions arising from their artistic practice (DG-10). Manxin subsequently distinguished among knowledge gaps, difficulties in connecting students’ questions with the course, and her anxiety about not being able to answer immediately. As she reflected, *“I had previously conflated these situations”* (DG-11).

The teachers then considered how theoretical concepts might be connected with the artistic practices valued by the new cohort. Juzi recalled overseas courses involving exhibitions, museum visits, and public art activities. Manxin adapted these memories to her own institutional context rather than reproducing them directly. She developed curatorial and cultural interpretation tasks through which students could use theoretical concepts in relation to visual form, audience, heritage, and artistic practice (LP2-04, ON-06).

Manxin interpreted the increased visible participation in some activities as evidence that the revised entry points were more accessible to this cohort. Nevertheless, the outcomes were uneven. Some groups used theoretical concepts in developing their interpretations, while others concentrated mainly on completing roles and producing the required outputs. Under time pressure, Manxin also returned to providing direct answers. She reflected, *“When I saw that time was running out, I returned to giving answers quickly. I thought a year of reflection would make me more patient, but under pressure I still took over the discussion”* (DG-13). Juzi cautioned that this was not simply a regression, but neither should every recurring difficulty be interpreted as recursive development, since some teaching habits had not completely changed (DG-13).

These experiences showed that a teacher could occupy the learner, explorer, and pedagogical enactor positions simultaneously or move among them as conditions changed. Re-entering the learner position did not mean returning to the original starting point. Manxin explained, *“I remained uncertain, but I could distinguish different kinds of problems and recognize that teaching had to change with the student group”* (DG-15). Juzi likewise continued to revise the meanings of her earlier teaching through engagement with Manxin’s current decisions. For both teachers, professional learning involved continued movement among *“action, doubt, interpretation, and renewed action”* rather than a linear progression from learner to pedagogical enactor (DG-15).

## 5 Discussion

### 5.1 Recursive identity negotiation through dialogic reflection

In response to the first research question, the findings identified three recurring identity positions: learner, explorer, and pedagogical enactor. These positions describe how the two teachers interpreted uncertainty, examined pedagogical alternatives, and translated developing understandings into classroom decisions. They did not constitute a linear progression toward an accomplished identity (Antera and Teräs, 2024; Christensen and Weissmann, 2025). Both teachers moved recursively among them, and more than one position could be present within the same teaching episode.

Taken together, these patterns are consistent with post-structural understandings of teacher identity as relational, situated, and continually negotiated rather than as a stable professional category (Hall, 1992; Butler, 1990). In the present study, this negotiation took shape through sustained dialogue between two returned teachers. Juzi’s retrospective experiences helped Manxin reconsider current classroom events, while Manxin’s experiences prompted Juzi to reinterpret assumptions underlying her earlier teaching. Dialogue therefore operated not simply as mentoring or advice but as a relational process through which both teachers’ accounts remained open to revision (Guo and Sidhu, 2024).

The learner position became visible when the teachers reconsidered immediate explanations of classroom difficulty. Manxin initially connected student silence with inadequate teaching ability, while Juzi had previously attributed similar difficulties partly to students’ dependence on teacher direction. Their dialogue complicated both interpretations by distinguishing observable classroom participation from possible explanations involving task design, preparation time, public expression, waiting time, and previous learning experience. These observations add classroom-level detail to research on how returned academics negotiate overseas experience within Chinese institutional and educational contexts (Ai and Wang, 2017; Qiu et al., 2024). In the present cases, prior learning histories remained relevant, but their significance was considered alongside instructional design and classroom relationships (Soong et al., 2021).

The explorer position involved provisional experimentation rather than the straightforward transfer of overseas pedagogical practices (Ai and Wang, 2017; Wang et al., 2021). The teachers reconsidered explanation, scaffolding, grouping, and project design according to their pedagogical functions. In particular, their experiences suggested that teacher guidance and student agency were not always experienced as opposing conditions. Explanation could close interpretation by supplying an expected answer, but it could also provide conceptual resources for further inquiry. Similarly, reducing teacher support did not necessarily generate autonomy when students lacked resources for formulating questions, organizing collaboration, or evaluating information (Heystek et al., 2025). This account complements professional-development research on teacher agency and learning by drawing attention to repeated interpretation and pedagogical redesign within everyday teaching (Wang et al., 2021; Vermunt et al., 2023; Liang et al., 2025).

The pedagogical enactor position described moments when developing interpretations became visible in coordinated decisions about content, support, participation, and classroom roles. It was not an endpoint indicating that professional uncertainty had been resolved. The teachers continued to encounter uneven participation, limited use of theoretical concepts, and a tendency to resume greater control under time pressure. Re-entering the learner position therefore did not represent either simple regression or the beginning of an identical cycle (Christensen and Weissmann, 2025; Sahling and De Carvalho, 2021). Earlier experiences remained available as resources, but new students and classroom conditions reopened questions about knowledge, authority, and pedagogical design. In these two cases, professional development involved recurrent negotiation across changing classroom conditions rather than movement toward a settled professional identity (Tucker, 2025).

### 5.2 Reflective practice as an interpretive Lens

**Figure 1:**
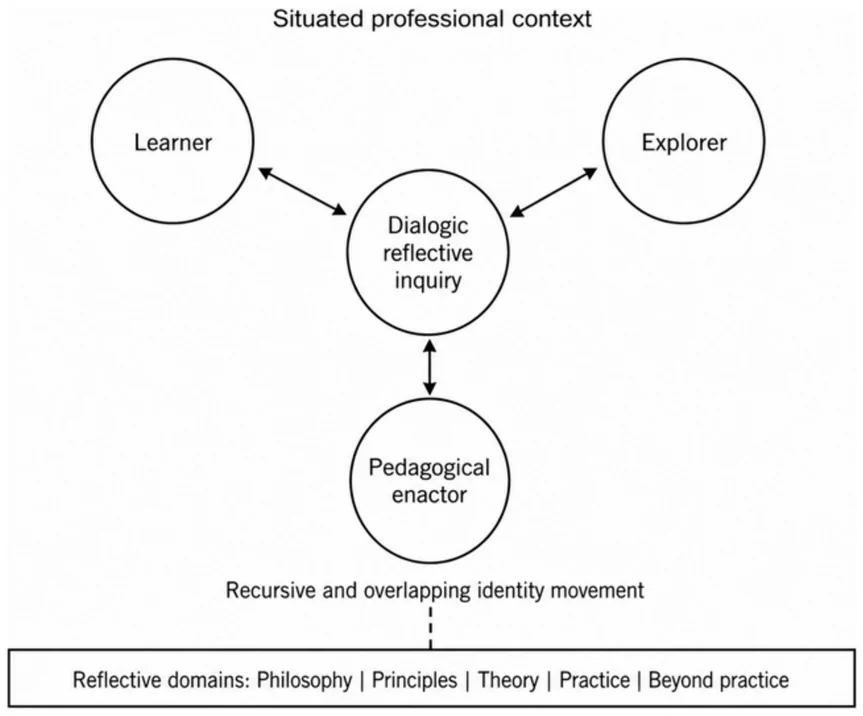
Dialogic negotiation of recursive teacher identity positions.

In response to the second research question, dialogic reflection connected identity negotiation with teacher learning and situated pedagogical enactment. Farrell’s (2020) framework provides a vocabulary for considering the concerns negotiated across the three identity positions. In this study, the five domains were used as interconnected interpretive lenses rather than as a sequence aligned with the positions. The identity positions and their recursive movement were developed from the dialogic data and are not components of Farrell’s framework. As represented in Figure 1, the domains offer interconnected lenses for examining the content of the teachers’ reflection without assigning a particular domain to each identity position.

Questions of philosophy appeared when the teachers reconsidered what art theory education should enable students to do. They continued to value interpretation, critique, and student agency, but became less likely to treat visible discussion as sufficient evidence that these purposes had been achieved (Tucker, 2025). Questions of principles concerned participation, trust, authority, and the conditions under which students could undertake public interpretation. Theory became visible in their reconsideration of project-based learning and student-centered teaching, particularly their recognition that these approaches required contextual adaptation rather than direct transfer (Wang et al., 2021; Heystek et al., 2025).

Practice involved observable changes to task sequencing, conceptual prompts, grouping, individual preparation, and role allocation (Vermunt et al., 2023). Beyond practice directed attention to how overseas educational histories, institutional expectations, disciplinary conventions, student cohorts, and immediate classroom conditions shaped pedagogical possibilities. Cultural materials were part of this wider context, but they were not treated as inherently accessible or educationally effective. Campus visual culture, regional art forms, curatorial practices, and intangible cultural heritage became objects through which theoretical concepts could be applied and questioned (Wang et al., 2025; Kang et al., 2025).

The analysis therefore distinguished the teachers’ recursive movement among identity positions from the concerns interpreted through Farrell’s (2020) domains. Questions within any of the five domains could arise across the identity positions and prompt further reconsideration, while the position occupied at a particular moment shaped what the teachers noticed and discussed. Figure 1 depicts this reciprocal relationship without assigning the positions a developmental order.

### 5.3 Implications for higher education teaching and teacher development

The findings suggest that universities employing returned teachers may benefit from providing sustained opportunities for the collaborative examination of classroom experience (Zhang et al., 2021; Bas, 2026; Karakose et al., 2026). Returned teachers may bring commitments developed through overseas education, but their professional learning involved more than either transferring these practices directly or abandoning them when students responded differently than expected. Peer dialogue, classroom observation, reflective writing, and critical friendship can support teachers in separating observation from interpretation, identifying competing explanations, and examining how pedagogical commitments operate under changing conditions (Ai and Wang, 2017; Sahling and De Carvalho, 2021).

Teacher-development programs can use Farrell’s (2020) five domains as prompts for inquiry without turning them into a prescriptive sequence. Teachers may examine their educational purposes, assumptions about participation and authority, interpretations of pedagogical models, classroom actions, and the institutional or cultural conditions surrounding those actions. The cases also indicate that professional uncertainty may reflect not only content-knowledge concerns but also relationships among task design, theory-practice connections, classroom interaction, and professional legitimacy (Christensen and Weissmann, 2025).

For art theory teaching, the findings support connecting theoretical concepts with students’ visual environments and cultural resources through structured interpretive tasks (Wang et al., 2025; Kang et al., 2025). However, culturally situated content can be incorporated without prescribing cultural identification or assuming that students will respond to it in the same way. Cultural heritage, campus visual culture, and regional art forms can instead be examined as contested objects involving selection, representation, audience, commercialization, and omitted perspectives. Such an approach creates opportunities for theoretical interpretation without predetermining what students should conclude.

The teacher-generated data indicated more visible participation in some redesigned activities, but they did not demonstrate corresponding changes in student learning, agency, or cultural identification. The implications therefore concern the teachers’ professional reasoning and pedagogical design rather than verified student outcomes. Within this boundary, the study shows how dialogic reflection can support contextually responsive teaching while keeping pedagogical interpretations provisional and open to further inquiry.

## 6 Conclusion

This study examined how two returned university art theory teachers negotiated their professional identities while reconsidering overseas pedagogical commitments in Chinese university classrooms. The analysis identified learner, explorer, and pedagogical enactor as overlapping identity positions rather than successive stages. Movement among these positions occurred as classroom encounters prompted uncertainty, dialogic questioning, provisional experimentation, and pedagogical action. Across changing classroom conditions, the teachers revisited these positions with understandings developed through earlier reflection and practice (Antera and Teräs, 2024; Christensen and Weissmann, 2025).

Through duoethnographic dialogue, both teachers revisited the assumptions that shaped their interpretations of classroom events. Juzi’s retrospective experiences informed Manxin’s examination of current teaching, while Manxin’s classroom experiences changed how Juzi understood her earlier decisions. Their exchanges presented professional learning as a relational process in which experience acquired new meanings through questioning, comparison, and reinterpretation (Norris and Sawyer, 2012; Guo and Sidhu, 2024).

For the two teachers, contextually responsive practice involved reconsidering how theoretical explanation, scaffolding, participation structures, and visual or cultural materials functioned for particular student groups (Wang et al., 2025). These cases suggest the value of sustained peer dialogue, observation, and reflective writing for returned teachers adapting educational experiences across contexts (Sahling and De Carvalho, 2021; Wang et al., 2021). They also indicate that cultural materials can be approached as objects of critical interpretation without assuming shared cultural responses or prescribing particular forms of identification. As the evidence was teacher-generated, these conclusions concern professional reasoning and pedagogical design rather than demonstrated changes in student learning, agency, or cultural identification.

The study is bounded by its focus on two female teacher-researchers working in related disciplinary and regional contexts. The teachers were also authors and analysts, and Juzi’s account relied partly on retrospective memory. The analysis did not include student interviews, student work, classroom video, or longitudinal evidence of learning, nor did it compare returned teachers with locally educated colleagues. Future research could examine whether similar identity positions and recursive movements are evident across disciplines, institutions, regions, genders, and career stages. Comparative studies incorporating student-generated and longitudinal evidence could also investigate how teachers’ reflective interpretations relate to classroom experience and learning without assuming that pedagogical redesign produces particular student outcomes.

## Data Availability

The original contributions presented in the study are included in the article/supplementary material, further inquiries can be directed to the corresponding author.
