# Supplementary material for: A Duoethnographic study of professional identity negotiation by two returned art theory teachers in Chinese higher education
Source: Front Psychol. 2026 Sep 1;17:1922669. doi: 10.3389/fpsyg.2026.1922669 (PMC13574694; doi:10.3389/fpsyg.2026.1922669)
Supplement: Supplementary file 1 [file Supplementary_file_1.docx]

**Appendix A.** Illustrative analytic progression from data sources to themes

| **Data source and representative data pattern** | **Initial descriptive and interpretive codes** | **Analytic category** |  | **Final theme** | **Later theoretical mapping** |
| --- | --- | --- | --- | --- | --- |
| In DG-04 and RJ-M02, Manxin experienced limited public responses as evidence that she might be unable to teach effectively. Juzi questioned the waiting time, preparation format, task wording, and distinction between observation and interpretation. ON-01 subsequently showed different levels of participation in individual writing, peer discussion, and whole-class speaking. | Experiencing silence as teaching failure; professional self-doubt; rapidly filling silence; competing explanations of participation; distinguishing visible silence from non-participation | Professional legitimacy challenged through competing interpretations of classroom silence |  | Entering a learner position through professional uncertainty | Philosophy; Principles; Practice |
| In DG-05, Manxin initially interpreted increased explanation as a retreat from student-centered teaching. Comparison with ON-01 and the revised LP1-05 shifted attention from the amount of teacher talk to whether explanation enabled students to apply and question concepts. | Equating explanation with teacher-centeredness; questioning the function of explanation; using concepts with new materials; redefining student-centered teaching | Functional reconsideration of explanation and pedagogical scaffolding |  | Entering an explorer position through dialogue and pedagogical experimentation | Principles; Theory; Practice |
| In DG-06 and RJ-J02, Juzi revisited her earlier decision to remove scaffolding and reconsidered her previous interpretation that students lacked autonomy. Comparison of LP1-09 with subsequent lesson plans and activity materials showed that Manxin retained support for question formation, collaboration, and source evaluation. | Withdrawing scaffolds too quickly; reinterpreting a remembered failure; shifting responsibility from student deficiency to task design; using another teacher’s memory to revise current action | Dialogic reconstruction of scaffolding and learner autonomy |  | Entering an explorer position through dialogue and pedagogical experimentation | Principles; Theory; Practice |
| ON-03, DG-07, and LP1-12 showed that familiar peer groups often began tasks more quickly but sometimes reproduced member dominance. Role rotation, individual preparation, and a designated questioner were subsequently incorporated into the activity design. | Reduced coordination cost; conditional effect of peer familiarity; unequal participation within familiar groups; structuring disagreement; revising group roles | Context-sensitive redesign of participation and collaboration |  | Entering the pedagogical enactor position | Theory; Practice; Beyond practice |
| In DG-10, Manxin initially attributed the difficulty of engaging first-year art majors in theoretical learning to her inadequate theoretical knowledge. Through DG-11, ON-05, and revisions to LP2-04, this difficulty was differentiated into content-knowledge gaps, theory-practice design problems, and anxiety about professional authority. | Generalizing difficulty as insufficient knowledge; separating visible participation from theoretical engagement; using practice problems as theoretical entry points; reclassifying professional uncertainty | Reorganization of the relationship among knowledge, pedagogy, and professional legitimacy |  | Moving recursively across identity positions under changing classroom conditions | Philosophy; Principles; Theory; Practice |
| ON-06 and DG-13 documented that Manxin could design a contextually responsive curatorial task while still providing direct answers when time pressure increased. Some groups used theoretical concepts substantively, whereas others treated roles and concepts superficially. | Contextualized pedagogical action; renewed teacher control under pressure; uneven enactment; coexistence of action and uncertainty; limiting claims about student outcomes | Overlap of identity positions and non-linear pedagogical change |  | Moving recursively across identity positions under changing classroom conditions | Practice; Beyond practice |
| Analytic memoranda and theme-review discussions compared chronological, strategy-based, Farrell-based, and identity-position structures. The chronological “teaching maturity” narrative was rejected, and the identity labels were revised from fixed stages to overlapping positions. | Comparing alternative thematic structures; resisting a linear growth narrative; checking theoretical circularity; identifying identity overlap and negative cases | Reflexive construction and revision of the identity model |  | Cross-theme analytic basis for the recursive identity interpretation | Farrell’s domains used after theme development |

**Notes.** The identifiers used in the findings and this appendix indicate the type and sequence of each data source. **DG** refers to a formally recorded reflective dialogue, with the following number indicating its chronological sequence. **RJ-M** and **RJ-J** refer to reflective journal entries written by Manxin and Juzi respectively. **ON** refers to Juzi’s classroom observation notes. **LP1** and **LP2** refer to lesson plans from the first and second semesters respectively. **ICH-01** refers to the teaching activity materials developed for the intangible cultural heritage project.
